# Human Microglia Respond to Malaria-Induced Extracellular Vesicles

**DOI:** 10.3390/pathogens9010021

**Published:** 2019-12-24

**Authors:** Smart Ikechukwu Mbagwu, Nils Lannes, Michael Walch, Luis Filgueira, Pierre-Yves Mantel

**Affiliations:** 1 Anatomy Unit, Department of Oncology, Microbiology and Immunology, Faculty of Science and Medicine, University of Fribourg, 1700 Fribourg, Switzerland; 2 Department of Anatomy, Faculty of Basic Medical Sciences, Nnamdi Azikiwe University, Nnewi Campus, Nnewi 435101, Nigeria

**Keywords:** microglia, extracellular vesicles, malaria-infected red blood cells

## Abstract

Microglia are the chief immune cells of the brain and have been reported to be activated in severe malaria. Their activation may drive towards neuroinflammation in cerebral malaria. Malaria-infected red blood cell derived-extracellular vesicles (MiREVs) are produced during the blood stage of malaria infection. They mediate intercellular communication and immune regulation, among other functions. During cerebral malaria, the breakdown of the blood–brain barrier can promote the migration of substances such as MiREVs from the periphery into the brain, targeting cells such as microglia. Microglia and extracellular vesicle interactions in different pathological conditions have been reported to induce neuroinflammation. Unlike in astrocytes, microglia–extracellular vesicle interaction has not yet been described in malaria infection. Therefore, in this study, we aimed to investigate the uptake of MiREVs by human microglia cells and their cytokine response. Human blood monocyte-derived microglia (MoMi) were generated from buffy coats of anonymous healthy donors using Ficoll-Paque density gradient centrifugation. The MiREVs were isolated from the *Plasmodium falciparum* cultures. They were purified by ultracentrifugation and labeled with PKH67 green fluorescent dye. The internalization of MiREVs by MoMi was observed after 4 h of co-incubation on coverslips placed in a 24-well plate at 37 °C using confocal microscopy. Cytokine-gene expression was investigated using rt-qPCR, following the stimulation of the MoMi cells with supernatants from the parasite cultures at 2, 4, and 24 h, respectively. MiREVs were internalized by the microglia and accumulated in the perinuclear region. MiREVs-treated cells increased gene expression of the inflammatory cytokine TNFα and reduced gene expression of the immune suppressive IL-10. Overall, the results indicate that MiREVs may act on microglia, which would contribute to enhanced inflammation in cerebral malaria.

## 1. Introduction

Malaria remains a global health burden, despite constant efforts channeled to its eradication. The disease is caused by the plasmodium parasite of different species, with *Plasmodium falciparum* (*P. falciparum*) being predominant and the most virulent [1]. According to the World Health Organization (WHO) [2], 216 million malaria cases have been recorded annually, with approximately 500 thousand deaths due to malaria infection. Sub-Saharan Africa bears the greatest burden of malaria infection. Although preventive measures have been promoted in the management of the disease, in addition to formulation of therapeutics, there have been limitations to achieve eradication due to widespread drug resistance as a result of parasite genetic factors [3,4,5,6].

Cerebral malaria (CM) is the most severe complication in both children and adults. CM often results in death or leaves survivors with neurological and cognitive impairment [7,8]. Some neuropsychiatric manifestations, which have been reported following CM, include visual hallucinations and anxiety symptoms [9], as well as impulsiveness and hyperactivity [10]. The precise mechanism involved in the pathogenesis of CM is still not well understood. However, various studies have described dysfunction of the blood–brain barrier (BBB) and secretion of proinflammatory cytokines to contribute to CM [6].

Recently, malaria-induced extracellular vesicles (EVs) have been reiterated as having a possible role in the pathogenesis of CM [11]. The effects of EVs has been well described in various biological processes, especially in intercellular communication [12] and regulation of the immune response [13]. They provide an insight into understanding the dynamics involved in host–pathogen interactions in various pathological conditions, including malaria. During malaria, EVs are released by various cell types, such as infected red blood cells, endothelial cells, platelets, and leucocytes [6]. In *in vitro* systems, plasmodium parasites cause modifications on the red blood cell membranes, which lead to the release of EVs. In the blood plasma of malaria patients, an increase in tumor necrosis factor alpha (TNF-α) was found to correlate with the level of extracellular vesicles. This also correlates with the severity of the disease [6,14,15].

The mechanism involved in the release of EVs derived from malaria-infected red blood cells (iRBCs) is through the haem–oxidative stress pathway [14]. EVs derived from plasmodium-infected red blood cell (MiREVs) are immunogenic and can influence systemic inflammation [16]. They are also known to promote antigen presentation and T cell stimulation [6,16,17]. We have reported that MiREVs contain small regulatory human and plasmodial RNAs, which are readily transferred to various cell types, including human bone marrow-derived endothelial cells, implying their role in host–pathogen interactions [18]. Human monocytes and neutrophils are also activated *in vitro* by MiREVs [6,19]. The internalization of *P. falciparum* DNA-containing MiREVs by monocytes showed that malaria DNA is capable of facilitating stimulator of interferon genes (STING)-dependent sensing to promote disease virulence [20]. Production of inflammatory cytokines and chemokines have also been implicated in *in vitro* cultures of monocytes stimulated with MiREVs [6].

EVs produced during malaria infection are known to cause vascular activation, as reported in Malawian pediatric CM patients with increased cell-specific extracellular vesicles [21]. The vascular mechanism involved in CM requires alterations in the cerebrovascular endothelium, apoptosis of the cerebral microvascular endothelial cells, upregulation of adhesion molecules such as Intercellular Adhesion Molecule 1 (ICAM-1), and parasite sequestration on the vascular endothelium. It has been shown that MiREVs are capable of disrupting barrier functions of human endothelial cells [19]. The immunological responses have been described to involve the production of pro- and anti-inflammatory cytokines, which are responsive to the malaria antigens [22]. In addition, genetic polymorphism in tumor necrosis factor alpha (TNF-α) and interleukin-10 (IL-10) has been implicated in the pathogenesis of CM [23,24,25,26,27,28,29,30,31,32]. The vascular dysfunction of the BBB emanating from these processes can cause the migration of substances from the periphery into the brain, and vice versa. Furthermore, it can also lead to the activation of other brain cells, such as the microglia, which would in turn foster the progression of the disease. This is supported by a murine model of cerebral malaria, which indicates the activation of microglia between 2 to 3 days of malaria infection [33]. EVs derived under different conditions from various cell types have been described to interact with microglia and the brain [34]. EVs from lipopolysaccharides (LPS)-challenged mice induce neuroinflammation [35]. The uptake of MiREVs by cells of glial cells has only be demonstrated in astrocytes and not in microglia [33]. Therefore, in our study, our aim was to investigate the uptake of MiREVs by human microglia cells and their cytokine response. 

## 2. Materials and Methods

### 2.1. Plasmodium falciparum In Vitro Culture

The 3D7 *P. falciparum* strain was used for this study. The parasites were kept in fresh type 0+ human erythrocytes suspended at 2% hematocrit in HEPES-buffered RPMI 1640 (Sigma, St. Louis, MO, USA), containing 10% (w/v) heat inactivated human serum, 0.5 mL gentamycin (Thermofischer-Gibco, Waltham, MA, USA), 2.01 g sodium bicarbonate, and 0.05 g hypoxanthine at pH 6.74. Prior to culture, the complete medium was depleted from extracellular vesicles and debris by ultracentrifugation at 100,000× *g* for 1 h before the culture. The parasite cultures were maintained in a controlled environment at 37 °C in a gassed chamber at 5% CO_2_ and 1% O_2_. 

### 2.2. Purification of EVs Derived from Malaria-Infected Red Blood Cells(iRBCs)

EVs from iRBCs were isolated from cell culture supernatants as described by Mantel et al. [19,36]. First, 15 ml of cell culture supernatants of *Plasmodium falciparum*-infected RBCs were collected. Cells and cellular debris were removed from the supernatant by centrifugation at 600× *g*, 1600× *g*, 3600× *g*, and, finally, 10,000× *g* for 15 min. To further concentrate the EVs, the supernatant was filtered through a Vivacell 100 filter (100 kDa molecular weight cut off; Sartorius). Then, the concentrated supernatant was pelleted at 100,000× *g*, the pellet resuspended in PBS and layered on top of a 60% sucrose cushion and spun at 100,000× *g* for 16 h. The interphase was collected and washed with PBS twice at 100,000× *g* for 1 h to yield EVs.

### 2.3. Generation of Human Microglial Cells

Human blood monocyte-derived microglia were generated from buffy coats of anonymous healthy donors obtained from the local blood bank (Blutspendedienst Bern, Switzerland) using a protocol adapted from Etemad et al. [37]. Human peripheral blood mononuclear cells (PBMC) were isolated from buffy coat after Ficoll-Paque density gradient centrifugation (1.077 g/L, Amersham Pharmacia Biotech AG, Dubendorf, Switzerland). Monocytes were enriched using positive selection of adherent cells, which were cultured at a concentration of 0.25 × 10^6^ cells/mL in RPMI-1640 GlutaMAX^TM^-I medium. Medium was supplemented with antibiotic/antimycotic and bioactive human recombinant granulocyte macrophage colony-stimulating factor (GM-CSF) (10 ng/mL), macrophage colony-stimulating factor (M-CSF) (10 ng/mL), nerve growth factor (NGF)-β (10 ng/mL), and CC chemokine ligand 2 (CCL2) (50 ng/mL) (all purchased from Miltenyi Biotech GmbH), at 37 °C and 5% CO_2_ for 7 days. Half of the medium was renewed after 3 days of culture.

### 2.4. Uptake of EVs Derived from Malaria-Infected Red Blood Cells and Confocal Microscopy

Purified MiREVs were labeled with a PKH67 green fluorescent labeling kit (Sigma-Aldrich), as has been previously described [19,38]. In brief, 4 μL of PKH67 was added to 400 μg of EVs in a total of 100 μL of diluent C, and incubated for 20 min at room temperature. Then, the label procedure of EVs was blocked with 200 μL of serum and ultra-centrifugated under the same conditions. After this step, the supernatant was discarded and the pellet of EVs was washed in 1 mL of PBS and ultra-centrifugated once more. The pellet containing PKH67-labeled EVs was resuspended in 400 µL of cell culture medium.

For confocal examinations, MoMi cells were cultured in Corning™ Clear Polystyrene 24-Well Plates (Corning, NY, USA). Then, 100 µg/ml of PKH67-labeled EVs was added for 4, 6, and 24 h of incubation. The cultures were then washed 3 times with PBS and fixed with cold (−20 °C) acetone for 5 min at −20 °C. DAPI staining was used to visualize nuclei. Cellular uptake of MiREVs was observed and recorded using Zeiss LSM 710 confocal laser microscope with an oil-immersion Plan-Apochromat 63× NA 1.4 objective (Carl Zeiss Microscopy GmbH), and three channels with lasers 488 nm (DAPI and PKH67), and 561 nm (Phalloidin). The confocal images were analyzed using Imaris software 8.0. Estimation of cells that internalized the MiREVs was performed using 15 images (*n* = 3). To visualize the uptake of MiREVs by MoMi cells in three-dimension (3D), we employed the method described by [39].

### 2.5. RNA Extraction and Gene Expression Studies in MoMi Cells

Total RNA was extracted from MoMi cells that were *untreated and treated with the conditioned media* (*supernatants containing MiREVs from Plasmodium* falciparum culture) using an RNAeasy Plus Mini Kit (250) (Qiagen, Germany). The concentration and integrity of total RNA was measured using a NanoDrop-1000 spectrophotometer (Thermo Scientific, Wilmington, DE, USA). An M-MLV Reverse Transcriptase kit (Promega, USA) was used to perform reverse transcription reactions performed with 1 μg total RNA. The cytokine-gene expression of TNF-α, IL6 and IL10 were detected by quantitative real time-polymerase chain reaction (qRT-PCR) using KAPA SYBR Green Fast (Kapa Biosystems. SA). 

Elongation factor-1 gene (EF1) was used as reference gene for relative quantitation using the 2^−Ct^ method. The following primers were used: TNF-α (forward: 5′-CAGCCTCTTCTCCTTCCTGAT-3′; reverse: 5′-GCCAGAGGGCTGATTAGAGA-3′). IL-6 (forward: 5′-CCACTCACCTCTTCAGAACGIL-3′; reverse: 5′-CATCTTTGGAAGGTTCAGGTTG-3′). IL-10 (forward: 5′-CGCATGTGAACTCCCTGG-3′; reverse: 5′-TAGATGCCTTTCTCTTGGAGC-3′). 

All experiments were carried out in a biosafety lab level 2. They were performed three times independently and in triplicates. Safety measures were applied accordingly. 

## 3. Statistical Analysis

All analyses were conducted with GraphPad Prism 8 (GraphPad Software). Variables were expressed as mean ± standard error of the mean (SEM). One-way ANOVA with appropriate post-comparison tests and two-tailed Student’s *t*-tests were used to analyze differences between populations where appropriate. A *p*-value < 0.05 was considered significant.

## 4. Results

MiREVs properties and composition have been recently investigated [18]. MiREVs uptake by endothelial cells has been reported by Mantel et al. [19] and shown to contribute to alteration in barrier properties of these cells, indicating MiREVs’ potential effect in the disruption of the BBB, which could lead to increased vascular permeability. This increased vascular permeability enhances the influx of substances, including circulating EVs into the brain, possibly leading to the stimulation and activation of surrounding brain cells such as the microglia. 

### 4.1. MiREVs Are Internalized by Microglia

**Figure 1:**
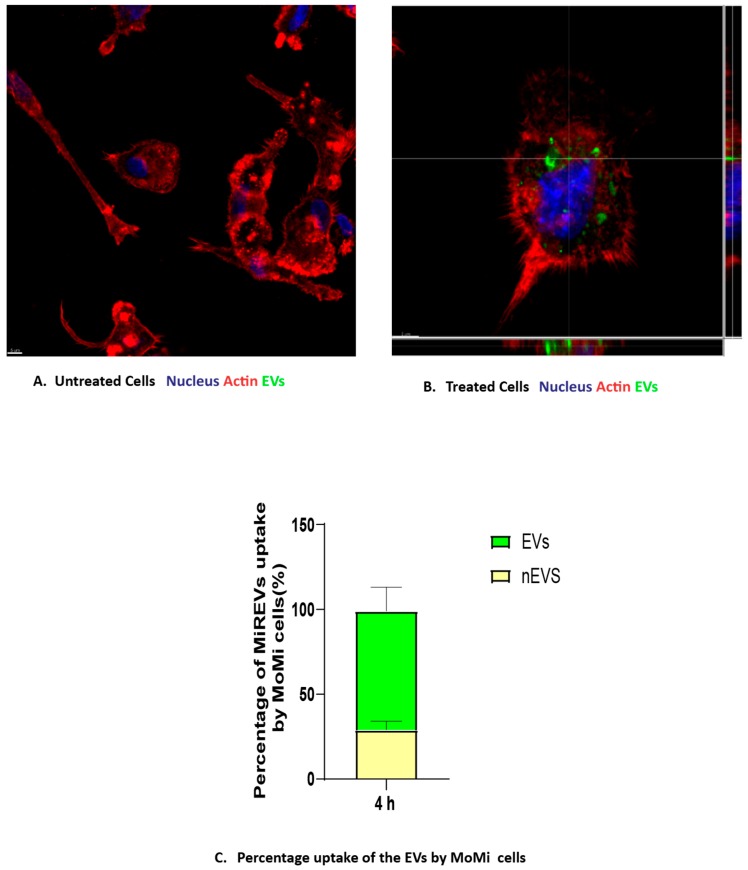
Cellular Internalization of malaria-infected red blood cell derived-extracellular vesicles (MiREVs) by monocyte-derived microglia-like cells. Monocyte-derived microglial-like cells (MoMi cells). The untreated cells (**A**) served as control sample, while the treated cells (**B**) were incubated with 100 μg of PKH67 fluorescently-labeled extracellular vesicles (EVs) at different time points. Lines indicate Z-stack. (**C**) Percentage of cells containing MiREVs (yellow colour represents cells not containing EVs(nEVs) while green colour represents cells containing EVs). The uptake was observed using confocal microscopy. The MoMi cells were stained for actin (phalloidin, red) and nuclei (DAPI, blue). *N* = 3. Scale bar, 5 μm.

We investigated the uptake of MiREVs by MoMi cells on the basis that circulating EVs can migrate into the brain during systemic inflammation [40] to establish a periphery–brain communication. In order to achieve this, we treated MoMi cells generated from the peripheral blood mononuclear cells with PKH67 fluorescent green dye-labeled MiREVs derived from red blood cells infected with *Plasmodium falciparum* at a concentration of 100 μg/mL for 4, 6, or 24 h. The uptake was assessed by confocal microscopy. Since the maximum impact was observed at the 4 h time point, the data shown are representative for the 4 h time point. We observed that the PKH67-labeled MiREVs were internalized by the MoMi cells and located within the cytoplasm of the cells (Figure 1A,B). The internalization of MiREVs occurred in about 70% of the MoMi cells (Figure 1C). Visualization of the uptake in 3D reconstructions showed that the MiREVs accumulated in the perinuclear region of the cells (Supplementary Material Movie S1). Furthermore, we also observed morphological changes in some of the MoMi cells to include granulations of the cytoplasm, formation of numerous pseudopodia, retraction of processes, and swelling of the cell body, indicating that the MiREVs had various effects on the cells (data not shown). Various mechanisms have been described to be responsible in the uptake of EVs by cells. To test what mechanism could be involved in the uptake of MiREVs by the MoMi cells, we co-incubated the cells for 4 h with actin filament inhibitors, such as Latrunculin A and Cytochalasin D, and observed that MiREVs’ internalization was completely inhibited in the presence of both inhibitors, indicating the importance of the actin cytoskeleton in the uptake process (data not shown).

### 4.2. Quantitative Cytokine-Gene Expression in Plasmodium Falciparum Culture Supernatant-Stimulated Microglia Cells

**Figure 2:**
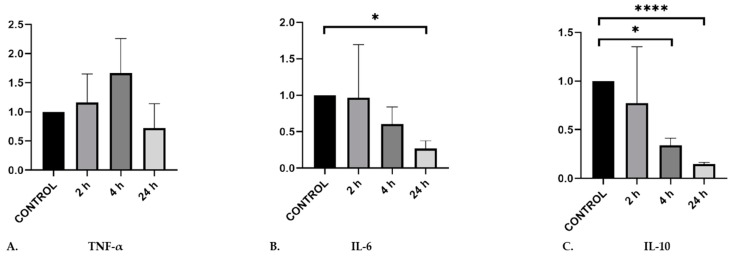
Gene expression of TNF-α, IL-6 and IL-10 by qRT-PCR in MoMi cells stimulated with supernatants from *Plasmodium falciparum* cultures. qRT-PCR results are normalized by the 2^−Ct^ method, using EF-1 as a reference and expressed as mean and fold induction over control (mean ± standard error of the mean (SEM); *n* = 3 independent experiments in triplicates; * *p* < 0.05; **** *p* < 0.001).

The activation of microglia by different mediators during malaria infection can contribute to neuroinflammatory processes during cerebral malaria [33]. Although there are variations in the reports on cytokine roles in cerebral malaria, the secretion of TNF-α has been well associated with neurological symptoms. On the other hand, the role of IL-6 in the development of cerebral malaria has not been well elucidated, as well as the role of IL-10, which may have double features during malaria infection. Therefore, we were interested to clarify whether MiREVs were able to induce gene expression of these cytokines in microglia and to infer, to an extent, the direction of immune response. To do so, we performed quantitative real-time PCR experiments to investigate the gene expression profile of these cytokines that have been associated with immune response in cerebral malaria. MoMi cells were stimulated with MiREVs derived from supernatants from *P. falciparum* cultures for 2, 4, and 24 h (*n* = 3), respectively. Gene expression of TNF-α was upregulated at 2 and 4 h and was downregulated after 24 h. However, this was not significant (*p* = 0.31), due to high variance (1.05). IL6 and IL10 were significantly downregulated in a time-dependent manner (IL6: *p* = 0.4967 between control and 24 h; IL10: p = 0.0280 between control and 4 h, and *p* < 0.0001 between control and 24 h) (Figure 2). Comparatively, there was only a statistically significant different between TNF-α and IL-10 at 4 h. These results indicate that MiREVs may induce expression of the inflammatory cytokine TNFα, but certainly reduce expression of the immune suppressive IL-10, which may contribute to the enhanced inflammation in cerebral malaria.

## 5. Discussion

In the central nervous system, microglia are the principal immune cells that defend the brain from pathogens and maintain cerebral homeostasis. The interaction between microglia and EVs from different cellular sources has been documented. Microglia have also been shown to internalize plasmodium-infected red blood cells in an *in vitro* malaria model, unlike astrocytes that were reported to have internalized the MiREVs [33]. However, this has been the first study involving EVs from malaria-infected red blood cells. Here, we show that human microglia take up MiREVs. The internalized MiREVs were found within the cytoplasm accumulating in the perinuclear region, similar to the uptake of MiREVs by mouse bone marrow-derived endothelial cells [19] and endothelial-derived extracellular vesicles uptake by endothelial cells [41]. This perinuclear localization of the internalized MiREVs is indicative of a programmed intracellular migratory or transport pattern, aimed at cargo delivery that would initiate gene regulation in the recipient cell nucleus [19,42]. 

The uptake of MiREVs also resulted in morphological changes in the phenotype of the microglia cells to include granulations of the cytoplasm, formation of numerous pseudopodia, retraction of processes, and swelling of the cellular body, suggesting microglial activation (data not shown). Mantel et al. [19] showed a time-dependent increase in the uptake of MiREVs in bone marrow-derived endothelial cells. In this study, the optimum uptake was observed at 4 h of treatment of MoMi cells with the MiREVs, as indicated by the number of cells found to contain the MiREVs. The degradation of the vesicles [43] and dispersion of the dye after this time could have resulted in a decrease in the number of cells containing intact EVs afterwards. The morphological changes are indicative of the M1-like microglial state, which is characterized by active phagocytosis and the induction of proinflammatory signals [44]. 

Uptake of EVs by microglia can be mediated by various forms of endocytosis, including micropinocytosis and phagocytosis [39,45]. These processes often involve receptor-mediated mechanism of uptake, in which actin plays a role [46]. The internalization of MiREVs by MoMi cells observed in this study appears to have involved membrane–receptor interaction, shown by the inhibition of the uptake of MiREVs in the presence of cytochalasin D and Latriculin A (data not shown). The action of these inhibitors is via actin polymerization [45]. The exact process involved in the uptake is not certain, as micropinocytic mechanism does not always involve a receptor–ligand interaction [46]. However, it is clear that this uptake is an active process. 

MiREVs are immunomodulatory and can mediate inflammation when they activate their target cells [11]. The secretion of proinflammatory cytokines, such as TNF-α, and immunomodulatory cytokines, such as IL10, has been well linked to the pathogenesis of CM and can, as such, compromise the integrity of the blood–brain barrier [1]. We also investigated the response of our microglia model to stimulation by parasite culture supernatants containing MiREVs. We found a significant downregulation in proinflammatory cytokine IL-6 and in the immunosuppressive anti-inflammatory cytokine IL-10. TNF-α was, however, upregulated (although statistically not significant). TNF-α and IL-6 are both proinflammatory cytokines, which are associated with the severity of malaria [47]. A counter-regulatory mechanism could have been responsible for the downregulation of IL-6, as observed in our study. This is also supported by the fact that TNF-α regulates IL-6 [48]. The upregulation of TNF-α indicates the response of the cells towards inflammation, which cannot be abrogated by anti-inflammatory IL-10 due to its low levels. This observation typically reflects an exacerbation of CM attributed to an imbalance in anti-inflammatory and proinflammatory cytokines [1]. 

The production of TNF-α by microglia in the brain can be sustained under the influence of interferon-gamma (IFN-γ) [49]. Proinflammatory responses mediated by TNF-α in the CNS trigger the pathogenesis of cerebral malaria by partly contributing to the induction of endothelial activation, increased blood–brain barrier permeability, and neuroinflammation [1]. Earlier investigations did not find evidence to support the role of IL6 in the pathogenesis of malaria [50]. This notion changed with evidence of IL-6 induction in pediatric cerebral malaria cases [7]. The production of IL-6 by microglia is specific for regulation of responses during the early phases of infectious states in the brain [51]. However, it tends to drive towards a proinflammatory or anti-inflammatory response, depending on the influence of other cytokines [52,53]. IL-10 is produced by microglia through Toll-like receptor-mediated stimulation following pathogen recognition receptor signaling [54,55]. Its production levels determine the state of the microglia and its consequent action. 

Our study succeeded in showing that there is an interaction between EVs from malaria-infected red blood and microglia. Microglia in the brain may interact with these EVs from infected red blood cells when they cross the BBB. This can be made possible when there is dysfunction in the vascular permeability of the BBB, initiated by the sequestration of malaria-infected red blood cells on cerebral microvasculature [56]. EVs have also been shown to foster sequestration of parasitized red blood cells. In addition to the vascular damage, there is also increased influx of leukocytes and other host- or parasite-derived materials, including circulating MiREVs into the brain, thereby stimulating and activating other cells, such as the microglia [57]. The direct migration of inflammatory EVs released by peripheral immune cells under the influence of systemic inflammation to target brain cells could be one possible mechanism for MiREVs to interact with the microglia [40]. When this happens, neuroinflammation driven by an EV-mediated periphery–brain communication could be established, as has been demonstrated in microglial uptake of plasma EVs [34] and EVs from LPS-challenged naïve mice [35]. 

There is variation in reports relating to the induction of cytokine responses in malaria and their effects. For instance, Kwiatkowski et al. [58] and Wilson et al. [59] reported that TNF-α levels corresponded to the severity of CM. On the other hand, elevated TNF-α levels in the cerebrospinal fluid of pediatric victims of malaria was only associated with neurological and cognitive deficits [60]. Also, elevated levels of TGF-β, TNF-α, IL-10, and IL-1β were found in cerebral malaria, while IL-2, IFN-γ, IL-5, IL-6, and IL-12 were only increased in mild malaria [1]. There is also variability in the response of IFN-signaling during malaria infection. The precise effect has not been clearly defined in humans, while the evidence of variability has been majorly drawn from the use of animal models, showing that it could either be beneficial or detrimental in malaria [61]. That notwithstanding, the implication of these observations suggests that these variations in cytokine induction could reflect the extent of virulence and pattern of clinical manifestation [62]. The differential expression of inflammatory cytokines in cerebral malaria is still a challenge that remains to be unraveled, as available results still need better characterization in defining its outcomes. 

## 6. Conclusions

Taken together, we have demonstrated that human microglia is capable of internalizing MiREVs and eliciting cytokine responses towards neuroinflammation. This interaction of human microglia with MiREVs suggests activation of the cells, which could result in several immunomodulatory responses acting towards neuroinflammation. However, the precise nature of the activation remains to be verified. It is needful to further characterize the mechanisms of uptake of MiREVs by microglia and the preceding responses, which would be beneficial in understanding the development of cerebral malaria. 

## Supplementary Materials

The following are available online at https://www.mdpi.com/2076-0817/9/1/21/s1.
